# Haste Makes Waste: There Is No Solid Evidence to Translate the Use of Stem Cells into Clinical Practice for Children with Autism Spectrum Disorder

**DOI:** 10.3390/brainsci12080992

**Published:** 2022-07-27

**Authors:** Antonio Narzisi

**Affiliations:** IRCCS Stella Maris Foundation, 56018 Pisa, Italy; antonio.narzisi@fsm.unipi.it

Increasingly, private clinics around the world offer stem cell therapy as a therapeutic approach for autism spectrum disorder (ASD) [1]. This phenomenon is extremely dangerous due to the fact that it is a practice that does not rest on solid scientific grounds and therefore risks illuding many families with respect to possible vaunted therapeutic effects.

While the use of stem cells for ASD could be potentially interesting field of research on the other hand the studies conducted to date are not characterized by a high degree of methodological rigor that enables their immediate translational impact [1].

The studies are characterized by a small sample size [2,3,4]; furthermore, they do not have a standardized and shared protocol of evaluation [2,3,4,5,6], do not describe a standardized method of treatment [4,5,7,8,9,10,11,12], and there are not robust data on the mid- and long-term effects of treatment [2,3,4,5,6].

In 2020, a randomized study [5] showed that the use of stem cells can be promising but was not associated with definite significant improvements in social skills or in reduction of autistic symptoms. For this reason, these authors conclude by emphasizing that more research is needed to determine whether the use of stem cells can be considered an effective treatment for some children with ASD.

In order to translate the results of scientific research to the clinical practice, it is also necessary to consider the risks of bias in the results obtained since they may be consistent among studies; however, all of these studies may be flawed [13].

Recently, the conclusions of a systematic review [14], which included a meta-analysis, stated that found no serious adverse events related to the stem cell therapy with children with ASD.

Unfortunately, this metanalysis also included uncontrolled and nonrandomized studies [9,10,12] that make their results rather unstable and therefore not immediately transferable to clinical practice.

In a more cautious way, Qu and colleagues [15] have conducted a meta-analysis, exclusively including randomized and controlled studies [2,3,4,5,6], focusing that although the area of research on the use of stem cells for ASD might be of great interest more studies are needed to systematically confirm the evidence of efficacy and safety of this therapy.

The take home message that needs to be considered is that, to date, the scientific evidence on the use of stem cells for the treatment of ASD is insufficient, and the paucity of registered clinical trials on this topic makes it impossible to suggest the use of stem cells in the field of clinical practice. The information on this topic to date is not immediately translatable to clinical practice, and we are still a long way from having solid data that would allow us to develop safe guidelines for the use of stem cells for ASD.

Compared to the current state-of-the-art on the topic, suggesting this type of clinical practice for ASD represents an unethical procedure. Future directions must involve continuing to study the use of stem cells for ASD, albeit through standardized methods that can provide us with solid data before suggesting its use in clinical practice. When the topic of debate concerns complex issues such as treatments of children, we have no other option but to be rigorous in the clinical methodology adopted. As clinicians and researchers, we have a duty to inform and protect our patients from therapeutic proposals that when not validated by research risk becoming quack practices.

## Data Availability

There are no data in this paper.
